# Measurable residual disease monitoring for patients with acute myeloid leukemia following hematopoietic cell transplantation using error corrected hybrid capture next generation sequencing

**DOI:** 10.1371/journal.pone.0224097

**Published:** 2019-10-28

**Authors:** Vidya Balagopal, Andrew Hantel, Sabah Kadri, George Steinhardt, Chao Jie Zhen, Wenjun Kang, Pankhuri Wanjari, Lauren L. Ritterhouse, Wendy Stock, Jeremy P. Segal

**Affiliations:** 1 Department of Pathology, Division of Genomic and Molecular Pathology, The University of Chicago, Chicago, Illinois, United States of America; 2 Department of Medicine, Section of Hematology/Oncology, The University of Chicago, Chicago, Illinois, United States of America; 3 Center for Research Informatics, The University of Chicago, Chicago, Illinois, United States of America; Universitat de Barcelona, SPAIN

## Abstract

Improved systems for detection of measurable residual disease (MRD) in acute myeloid leukemia (AML) are urgently needed, however attempts to utilize broad-scale next-generation sequencing (NGS) panels to perform multi-gene surveillance in AML post-induction have been stymied by persistent premalignant mutation-bearing clones. We hypothesized that this technology may be more suitable for evaluation of fully engrafted patients following hematopoietic cell transplantation (HCT). To address this question, we developed a hybrid-capture NGS panel utilizing unique molecular identifiers (UMIs) to detect variants at 0.1% VAF or below across 22 genes frequently mutated in myeloid disorders and applied it to a retrospective sample set of blood and bone marrow DNA samples previously evaluated as negative for disease via standard-of-care short tandem repeat (STR)-based engraftment testing and hematopathology analysis in our laboratory. Of 30 patients who demonstrated trackable mutations in the 22 genes at eventual relapse by standard NGS analysis, we were able to definitively detect relapse-associated mutations in 18/30 (60%) at previously disease-negative timepoints collected 20–100 days prior to relapse date. MRD was detected in both bone marrow (15/28, 53.6%) and peripheral blood samples (9/18, 50%), while showing excellent technical specificity in our sample set. We also confirmed the disappearance of all MRD signal with increasing time prior to relapse (>100 days), indicating true clinical specificity, even using genes commonly associated with clonal hematopoiesis of indeterminate potential (CHIP). This study highlights the efficacy of a highly sensitive, NGS panel-based approach to early detection of relapse in AML and supports the clinical validity of extending MRD analysis across many genes in the post-transplant setting.

## Introduction

Due to the high mortality rate and frequency of treatment failures, improved methods of disease status monitoring are clearly needed for acute myeloid leukemia (AML) patients during therapy. In particular, enhanced techniques for surveillance of low level measurable residual disease (MRD) following hematopoietic cell transplantation (HCT) are critical, as up to half of all such patients experience recurrence[1]. Better surveillance systems may improve prognostication and facilitate earlier therapeutic interventions, potentially preventing disease recurrence.

Standard-of-care methodologies for evaluating for recurrence in AML currently include morphologic assessment of the bone marrow (BM) and engraftment analyses using short tandem repeat (STR) polymerase chain reaction (PCR). Marrow histological analysis has variable sensitivity for recurrence, as regenerative blasts may confound accurate assessment. MRD flow cytometry for AML can require highly multiplexed analysis and is often complicated by variable sensitivity due to patient-specific marker expression profiles. These analyses can also be subject to inter-assay and inter-operator variability[2–5]. STR PCR assays are generally applicable to all HCT patients due to their use of common identity markers but are limited by a sensitivity for MRD of approximately 1–5%[6–9]. Notably, STR-based assays do not specifically measure recurrent disease but instead offer a percentage of recipient DNA as a surrogate measure for recurrence. This impairs specificity, as non-malignant recipient cell lineages may be present in various sample types and conditions without disease relapse[10].

To maximize sensitivity and specificity, methodologies that focus on low-level detection of oncogenic driver mutations are clearly preferable. Unfortunately, the development and incorporation of expanded low-level mutation detection technology into routine AML care has lagged due to a variety of technical and biological factors. Focal assays such as RT-PCR may be applied to individual genetic alterations. This, however, is a major limitation in a disease notable for a strikingly broad array of different potential oncogenic driver events across many genes. The advent of next generation sequencing (NGS) has recently permitted deeper/higher sensitivity analysis of single genes such as *NPM1* and *FLT3* [11]. In addition, broader MRD assessments of patients with AML beyond common markers is also possible [12]. However, using standard library preparation systems, NGS still suffers from relatively low specificity, resulting from PCR and sequencing error, necessitating higher variant allelic frequency (VAF) cutoffs[13,14]. Fortunately, this limitation can be circumvented by the incorporation of unique molecular indices (UMIs) into the library preparation in order to tag individual molecules and allow for proofreading following intentional over-sequencing [15–17]. Properly applied, this can dramatically reduce the inherent error rate of preparation and sequencing and allow mutation detection at VAFs of 0.1% or below. For hematological malignancies, these techniques have mainly been applied for detection of hotspot mutations in only a few genes, and only during the post-induction and peri-transplant phases to help guide transplant decisions [12,18–20]. These studies were primarily aimed at determining who should move more quickly to transplant or for prognosticating whether a transplant would be successful. In such scenarios, expansion of MRD analysis across many heme malignancy related genes has been complicated by the persistence of residual pre-malignant clonal mutations [12]. However, the purpose of this study is to assess the suitability of this technology for long-term screening surveillance after complete engraftment. We hypothesized that pre-malignant clones were unlikely to persist in such patients, and that the analysis could effectively be expanded to include essentially any mutated gene, thus making this a potentially powerful application for larger-scale NGS.

To investigate the effectiveness of expanded territory NGS for MRD detection in this setting, we developed and optimized a twenty-two gene hybrid-capture NGS panel covering commonly mutated genes in AML and other myeloid neoplasms. This assay incorporates UMIs and bioinformatic error-correction in order to achieve high sensitivity and specificity variant detection at or below 0.1% (1 in 1000). To test this system, we performed a retrospective comparison between our MRD NGS assay and our laboratory’s standard-of-care techniques for AML post-transplant monitoring (STR-PCR analysis and hematopathology bone marrow assessments), assessing this assay’s capacity for early detection using pre-relapse specimens that had previously shown no evidence of disease using our current methodologies. This analysis permitted a comparison of assay sensitivities and quantification of pre-recurrence lead-time using this method compared with our current standard-of-care approaches. The data set also provides insight into the kinetics of AML recurrence as well as the potential benefits and limitations of these techniques.

## Materials and methods

### Retrospective AML study design and sample collection

This study was approved by the Institutional Review Board (IRB) for Biological Sciences Division at the University of Chicago. The approval number is IRB16-0791. Consent was not obtained from the subjects. A waiver of informed consent was granted by the IRB. This was due to the minimal risk posed by the study as samples were archival and de-identified for the study.

**Fig 1:**
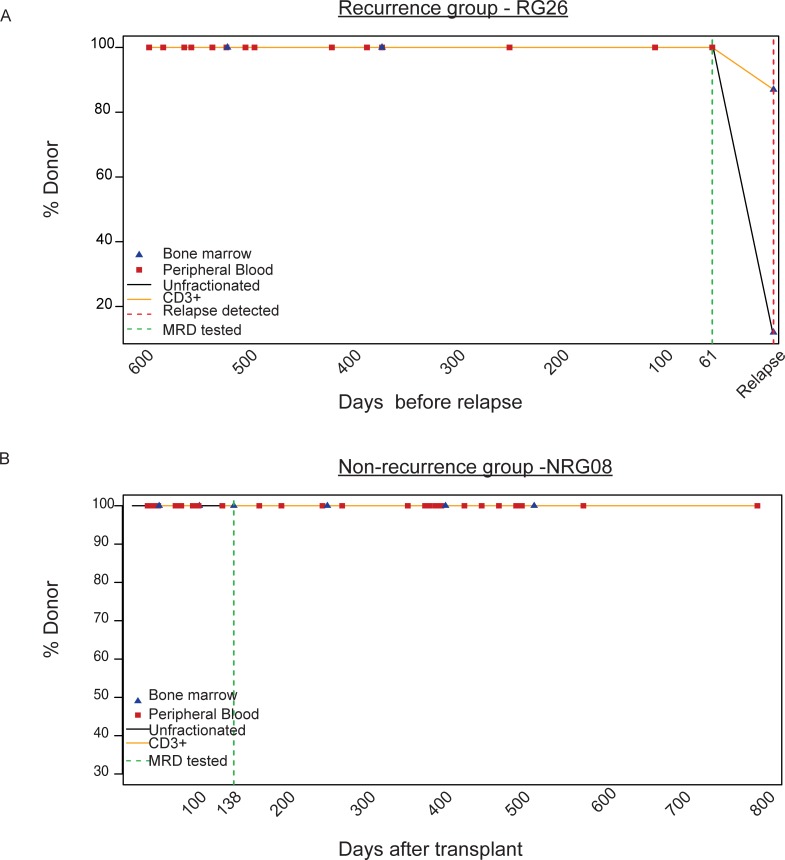
Retrospective AML study design. Post-transplant engraftment analysis via short tandem repeat (STR) PCR results are plotted as % donor over time. (A) Example of longitudinal engraftment analysis from one patient in the ‘recurrence group’ (RG29) is shown here. Time is shown as days prior to relapse. Red dotted line denotes the relapse time point, while the green dotted line indicates a fully-engrafted sample within the 20–100 day pre-relapse range. (B) Example of longitudinal engraftment analysis from one patient in the non-recurrence group (NRG08) is shown here. Time is shown as days post-HCT. The green dotted line shows a fully engrafted time-point that tested for MRD, with >3 months of prior and >2 years of subsequent follow-up with full engraftment.

To assess the performance and utility of NGS-based MRD detection in patients with AML following HCT, we designed a retrospective case-control study taking advantage of samples and data collected during routine clinical engraftment analysis of BM and peripheral blood (PB) using STR PCR. To identify patients suitable for inclusion in the study, we reviewed our results from the University of Chicago Medicine (UCM) Molecular Diagnostics Laboratory post-transplant engraftment testing from 2014–2018, with approval from the University of Chicago Institutional Review Board. Two sets of patients were included in the study if they possessed mutations trackable with the 22 gene MRD panel (Table 1): a recurrence group (RG) and a non-recurrence group (NRG)(Fig 1). Patients who relapsed after HCT were included in the RG if additional PB/BM samples were available from these patients which were collected from 20–100 days prior to recurrence and which showed no evidence of disease by STR PCR (Ampflster Profiler Plus or Identifiler Plus, Thermo Fisher Scientific, Waltham, MA) or hematopathology histological assessment. NRG patients were those whose AML has not been observed to relapse to-date. Samples selected from NRG patients were those showing complete engraftment by STR PCR and/or bone marrow histology (and standard flow cytometry analysis, if available) with at least 3 months of prior and 9 months of subsequent results also showing complete engraftment. In total, the RG group included 46 specimens (28 BM and 18 PB) from 30 patients, and the NRG group included 12 samples (5 BM and 7 PB) from 9 patients. RG patients ranged in age from 22 to 71 (median = 51 years), with an average of 2.5 trackable mutations per patient. NRG patients ranged in age from 18 to 68 (median = 61 years), and had an average of 1.8 trackable mutations per patient. Patient characteristics are listed in S1 Table.

**Table 1:** The genes and exons covered in the NGS MRD panel.

Gene	Transcript	Exons
*ASXL1*	NM_015338	12
*BRAF*	NM_004333	15
*CALR*	NM_004343	9
*CSF3R*	NM_000760	14,17
*DNMT3A*	NM_022552	23
*FLT3*	NM_004119	13–15,20
*IDH1*	NM_005896	4
*IDH2*	NM_002168	4
*JAK2*	NM_004972	14
*KIT*	NM_000222	8,17
*KRAS*	NM_033360	2,3
*MPL*	NM_005373	10
*MYD88*	NM_002468	5
*NPM1*	NM_002520	11
*NRAS*	NM_002524	2,3
*RUNX1*	NM_001754	4–9,
*SETBP1*	NM_015559	4
*SF3B1*	NM_012433	14–16
*SRSF2*	NM_003016	1
*TP53*	NM_000546	2–12
*U2AF1*	NM_006758	2,6
*WT1*	NM_024424	5–10

### Establishment of trackable variants for RG and NRG patients

To establish a set of trackable variants for each patient, DNA from recurrence samples from RG patients and pre-transplant samples from NRG patients were sequenced using one of two CLIA validated NGS panels in use in our laboratory, a 54 gene custom amplicon myeloid panel (OncoHeme) or the 1,213 gene pan-tumor OncoPlus panel [21]. Both panels cover all coding regions contained within the 22 gene MRD panel. Mutations from these samples present at VAF >5% and categorized as pathogenic by a pathologist were used as trackable mutations. Only patients that possessed mutations trackable by our heme MRD assay were selected.

### Capture-based NGS MRD assay

**Fig 2:**
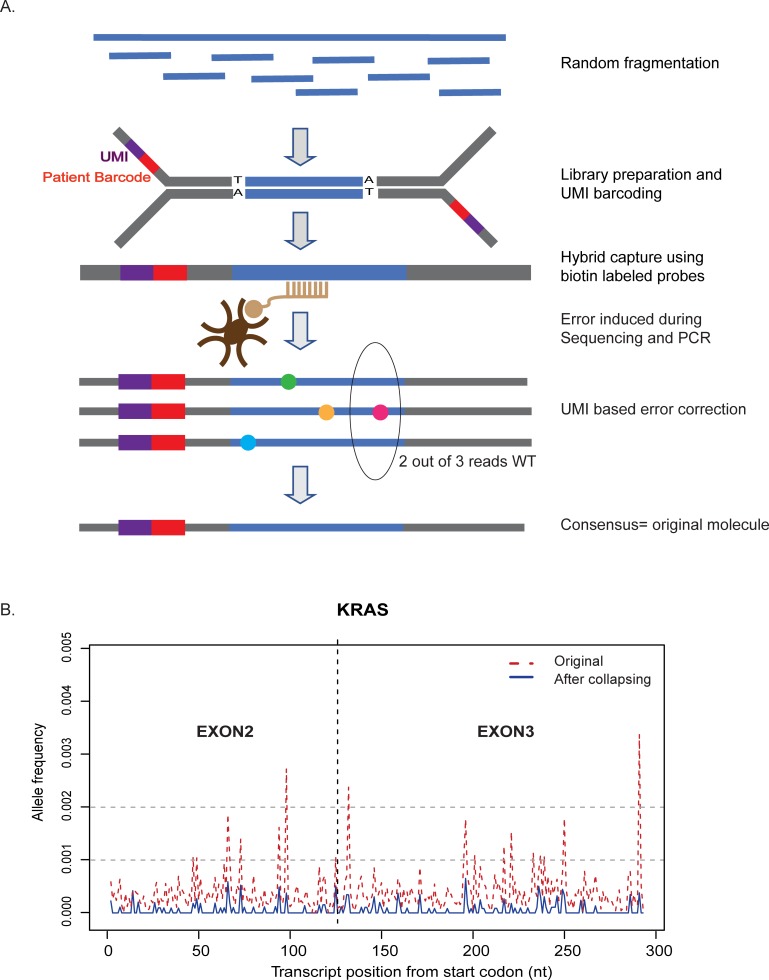
Error-corrected hybrid-capture NGS assay for MRD detection. (A) Each DNA molecule is tagged with a unique molecular barcode and patient barcode. DNA-seq libraries are prepared, captured for the regions of interest and sequenced. The UMIs are used for correction of errors introduced during PCR and sequencing. (B) UMI-based proofreading reduces error-associated noise. *KRAS* exons 2 and 3 are shown, highlighting reduction in background error rate in a representative sample.

To perform mutation-based surveillance for myeloid disease patients in our laboratory, we created a hybrid capture panel incorporating UMIs and associated custom bioinformatics software to enable correction of PCR/sequencing associated errors (Fig 2A). Briefly, DNA from PB/BM was quantified with Qubit reagents (Thermo Fisher Scientific, Waltham, MA). 400 ng was subjected to random ultrasonic fragmentation (Covaris M220, Woburn, MA) and library preparation using the KAPA HTP kit (KAPABiosystems, Wilmington, MA). Fragmented DNA was end-repaired and A-tailed followed by adapter ligation and PCR amplification (6 cycles). The adapters used in this assay included 6bp unique molecular identifiers (UMIs) adjacent to the patient barcode (IDT, Coralville, IA). 500 ng each of four libraries were pooled and subjected to 16hr hybridization and capture using 116 individual 120-mer biotin-labeled probes targeting the desired territory of 22 genes (total territory = 13.92 kb) (IDT, Coralville, IA). The captured pool was amplified for 13 cycles, and 2 ng of the capture pool was subjected to a second 4 hr hybridization with the same probe set to maximize on-target rate. At least 16 libraries were sequenced per flow cell on a HiSeq 2500 using rapid run mode (2x100bp) to obtain >15 million paired-end reads. With this amount of sequencing we were able to consistently obtain >99% mapping, >95% on-target rates, and >10,000 collapsed median depth at positions of interest.

### Informatics analysis

Custom pipelines were written to analyze the data on a high-performance computing cluster (Center for Research Informatics). Sequencing data were first aligned to the hg19 human reference genome, followed by aggregating into groups ideally representing individual original molecules based on both the ‘start’ and ‘end’ position of the read as well as the UMI sequences. Consensus sequences were derived from each read group that included 3 or more reads (to provide adequate proof-reading), with the consensus sequencing assigned to majority calls among the group at each position. If no consensus was reached among component reads at a given position, the consensus base call was defined as “N”, an undetermined nucleotide that would not participate in downstream depth statistics or variant calling.

Variant calling was performed on realigned data following consensus merging, using a previously published in-house variant summarization software (PileupAnalyzer) that operates on the output of Samtools mpileup [22]. Variants were annotated with Alamut Batch v1.4 software (Alamut, Rouen, France). For each patient, variant calls from pre-relapse samples were compared against that patient’s previously established list of trackable mutations (see above).

### Statistical analysis

Fisher’s exact test was done on the sample set to determine the association between MRD test results and relapse of AML. Mann-Whitney non-parametric test was conducted to test the difference in outcomes between the RG and NRG groups. P values were considered significant at 0.01.

Data Sharing Statement: Data cannot be shared publicly because of human subject clinical data. Data are available from the University of Chicago IRB office for researchers who meet the criteria for access to confidential data. For original data, please contact the Director of Regulatory Compliance for Human Subjects at the Office of Clinical Research, Millie Maleckar (mmalecka@bsd.uchicago.edu). The data set should be identified as Segal_MRA dataset.

## Results

### Clinical applicability of the NGS MRD assay

To determine the clinical utility of the designed panel for surveillance of patients with AML, we performed a retrospective analysis of NGS profiling results to determine its expected utility for AML patients. Since 2016, we have performed clinical sequencing with our 1,213 gene OncoPlus panel for 242 AML patients with adequate specimens (>10% blasts), of which 214 patients (88.4%) had mutations interpreted as pathogenic that fell within the territory of the 22 genes in the MRD panel (Table 1). Those 214 patients had a total of 743 trackable mutations within the panel territory, equating to a mean of 3.2 trackable mutations per patient. The majority of the patients without a trackable mutation were cases with recurrent cytogenetic abnormalities, which tend to lack other common driver mutations [23].

### Establishment of clinical and analytical specificity using samples from non-recurrence patients

Incorporation of UMIs and error-correcting bioinformatics algorithms into the assay workflow led to a marked reduction in false-positive noise across the assay territory (representative example of exon 2 and 3 of *KRAS* shown in Fig 2B). In order to establish the specificity of MRD analysis, we tested all collected NRG samples (7PB and 5BM from 9 patients) in remission for at least 1 year post-SCT using the MRD panel. At the well engrafted time-point tested, no trackable mutations were found in the NRG samples at or above the desired cut-off of 0.1% VAF (S2 Table), with no presence of indels at any observable VAF.

**Fig 3:**
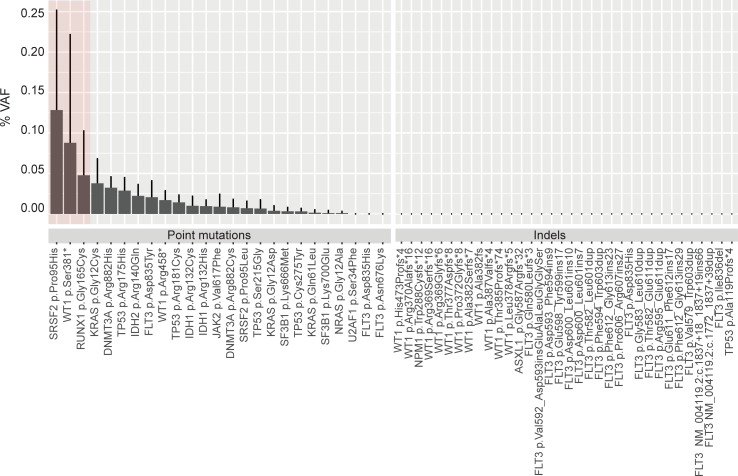
Specificity of the MRD assay. All trackable mutations included in this study (at relapse for RG and diagnosis for NRG, total = 59) were queried in the NRG samples. Pathogenic variants are listed at protein level change whenever possible. In the few instances where the protein change could not readily defined (e.g. *FLT3* ITDs involving an intron/exon boundary), RNA level change along with the transcript used is provided. The average VAF detected in the negative control (NRG) is shown in the histogram plot with standard deviation error bars. Only 3/59 variants were detected at or above 0.1% VAF, the desirable limit of detection. These variants are highlighted in a red box and were excluded from further analyses.

To examine the assay more broadly for technical specificity, we evaluated levels of false-positive noise associated with every trackable mutation across both the RG and NRG sample sets (Fig 3). Of these 59 combined pathogenic mutations (listed in S3 Table), 56/59 were clear of false-positive noise above 0.1% in these samples and thus were suitable for downstream analysis of RG samples. Three mutations (chr17:74732959 G>T, *SRSF2* p.Pro95His; chr11:32417910 G>T,*WT1*p.Pro95His and chr21:36252869 C>A, *RUNX1* p.Gly165Cys) showed elevated levels of artifactual noise across multiple samples and were excluded from downstream MRD analyses. These 3 mutations were all C>A or G>T changes, possibly associated with DNA oxidation accumulating in our samples over long-term storage[24]. Unsurprisingly, the residual errors still present after UMI proof-reading in these samples were substantially enriched for point mutations (which are common polymerase errors), whereas extremely clean signal was seen for indels. As a result, for analysis of the recurrence samples for the 56 remaining trackable mutations, we set detection thresholds of 0.1% VAF for point mutations and 0.001% VAF for indels.

### Retrospective analysis of pre-recurrence samples

**Fig 4:**
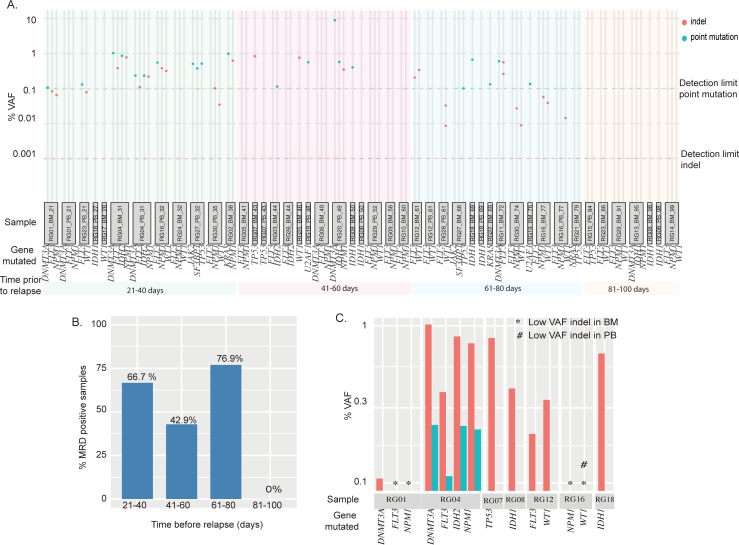
Early detection of relapse in RG patient samples using error-corrected NGS. (A) Shown are the results from MRD testing of all RG samples collected <100 days before relapse. Each sample ID (gray box) is identified with RG number, source (PB or BM) and days before relapse. VAF cutoffs of 0.1% and 0.001% were used for point mutants (green dotted line) and indels (red dotted line). Genes with trackable pathogenic variants detected at relapse are listed for each sample. The samples are grouped into four bins (21–40, 41–60, 61–80, and 81–100 days prior to relapse) and color-coded as green, pink, blue and orange, respectively. (B) Summary (bar plot) of MRD detection rate for samples from each bin (21–40, 41–60, 61–80, and 81–100 days) (C) VAF of trackable mutations from time-matched PB and BM samples (7 patients).

To determine the potential efficacy of MRD monitoring in the post-transplant setting, we tested all of the RG PB/BM DNA specimens previously analyzed as negative for disease in our laboratory at time points 20–100 days prior to recurrence. The 47 tracked mutations for this group were spread across 12 genes (*DNMT3A*, *FLT3*, *IDH1*, *IDH2*, *JAK2*, *KRAS*, *NPM1*, *NRAS*, *SF3B1*, *TP53*, *U2AF1*, and *WT1*), and included 38% point mutations and 62% indels (summarized in S2 Table). A patient’s sample was considered MRD-positive if any of that patient’s trackable mutations were identified. In this sample set, we successfully identified MRD in at least one pre-relapse time-point in 18/30 patients (60%). Detected VAFs ranged from 8.85% to 0.0014% (Fig 4A).

We applied Fisher’s exact test to our data to ask whether MRD positivity is associated with relapse. A calculated Fisher’s exact test statistic of 0.0016 (significant at p-value < 0.01) suggests that there is a statistically significant association between MRD positivity and eventual relapse. Further, A Mann-Whitney U test was conducted to test the difference between the VAFs of RG and NRG groups. The U test statistic was calculated as 280.5 with a z-score of 4.29462 and a p-value <0.001.

To better understand the time-dependence of MRD detection, we evaluated MRD detection power relative to lead-time before relapse. We were able to detect MRD in 67% samples when the last remission sample was collected 21–40 days (8 out of 12 samples) prior to relapse. 43% of samples collected 41–60 days prior to relapse (6 out of 14 samples) showed detectable mutations, as well as 77% of samples collected 61–80 days prior to relapse (10 out of 13 samples) (Fig 4A & 4B). Several patients in the 61–80 day group had indel mutations that were picked up at very low MAFs, which may explain the unexpected higher MRD detection rate in the 61–80 day group. We were unable to detect MRD in any samples collected 80 days or more prior to relapse (n = 7).

To understand if the type of input (BM or PB) had an effect on MRD detection rate, we calculated the MRD detection rate in PB and BM samples separately. Of the 46 samples analyzed for MRD, 28 were BM and 18 were PB. 16 of the 28 BM (53.6%) and 9 of the 18 PB (50%) samples tested MRD positive. This observation suggested that PB and BM samples perform similarly. However, previous reports have shown differences in signal between PB/BM [25–28]. It might be expected that early emergent disease would be easier to detect in BM vs. PB due to the cellular make-up of both specimen types and potential for delayed mobilization to the periphery. To investigate this, we examined date-matched PB/BM pairs available within our sample set (n = 7 pairs), comparing detection ability and mutation VAFs. In this group, BM samples showed significantly higher detection ability and mutation VAFs compared with PB (Fig 4C).

### Clinical utility across expanded gene sets in the post-HCT setting

**Fig 5:**
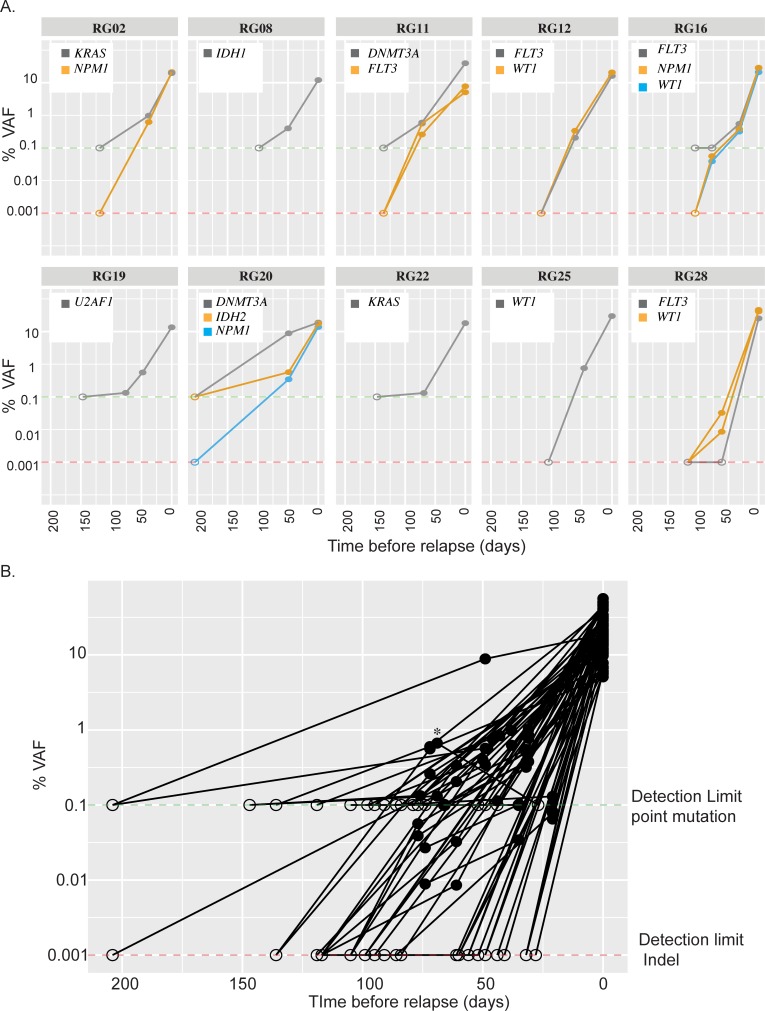
All trackable mutations can be used as MRD markers in post-transplant setting. **(**A) Shown are 10 MRD positive patients where additional fully-engrafted samples (via STR PCR/histology) were available in the > 100 day time-period prior to relapse. The VAF of the trackable pathogenic variants are followed over a time period of 0 to 220 days prior to relapse. In all 10 cases, we were unable to detect trackable variants at >100 day time points. (B) VAF of all trackable variants from all MRD-positive RG samples starting at relapse to 220 days prior. All pathogenic variants detected at relapse diminished over the course of time, with no variants detected at >80 days. In both Fig 5A and 5B, VAF cutoffs of 0.1% and 0.001% were used for point mutants (green dotted line) and indels (red dotted line) respectively and data points where %VAF was below the detection limit is plotted at detection limit but denoted with open circles while closed circles represent the measured %VAF.

After chemotherapy, patients with AML may continue to show evidence of pre-malignant clones or clonal hematopoiesis of indeterminate potential (CHIP) that does not readily portend relapse [12].These genes (DNMT3A, TET2, ASXL1, etc.) are of questionable utility as MRD markers in the post-chemotherapy setting [29–32]. However, in the post-transplant setting, we hypothesized that all trackable markers may have value, given the nature of a patient fully-engrafted with donor cells. To ascertain the clinical utility of MRD testing across expanded gene sets in the post-transplant setting, we tested additional samples from 10 MRD-positive RG patients, at fully engrafted timepoints between 100–210 days prior to relapse, as assessed by STR PCR/histology. A total of 3 PB and 7 BM samples were tested from these patients, and no pathogenic mutations were detected in any specimen (Fig 5A). Mutations tested at these time-points were in AML-specific genes (e.g. *NPM1*, *FLT3*) as well as genes commonly associated with mutations in CHIP (e.g. *IDH1/2*, *DNMT3A*, and *TP53*). Thus, variants in all genes tested appear to increase in VAF and become detectable only very close to relapse, permitting their effective use in MRD monitoring (Fig 5B).

## Discussion

Current standard-of-care approaches for post-transplant disease surveillance in AML are sub-optimal from the standpoint of cost, sensitivity and patient experience. Each BM biopsy entails substantial costs and discomfort for the patient [33], yet typical algorithms include serial biopsies, including histological, flow cytometric, and STR-PCR evaluations. In this study, we show that a straightforward, targeted error-corrected NGS capture panel can be used to perform surveillance for MRD in the post-transplant setting at an analytical sensitivity of 0.1% and below. In this retrospective analysis intended to mimic real-world use, this system performed substantially better than our current standard-of-care methodologies for detection of MRD. We were able to detect patient-specific mutational evidence for MRD in 62% of all samples collected 20–80 days prior to relapse which had all previously tested as negative for disease by a combination of STR PCR and standard hematopathology bone marrow analysis. However, in the remaining 38%, we were unable to find earlier evidence of relapse. It is likely that this reflects the variable kinetics of AML from patient to patient, with some patients experiencing more rapid clonal expansions during relapse. For the most rapidly recurring patients, it may be that future assays with further improved analytical sensitivity may be required in order to substantially advance detection lead-time. It should be noted that mutational evidence of disease was clearly present in all patients at their relapse dates, even by low sensitivity NGS profiling. Thus, implementation of this or a similar MRD assay into surveillance algorithms would be expected to substantially increase relapse detection lead-time in most patients by as much as three months, while at worst showing no improvement in lead-time.

A key feature of this assay is the incorporation of many genes into a comprehensive MRD panel, which allows for surveillance of a wide patient population. Compared with piecemeal testing of *NPM1*, which is mutated in approximately 35% of AML patients, we estimated this panel would be effective for more than 88% of patients seen in our laboratory. With the cost of sequencing continuing to decrease over time, additional territory will be easily added to panels at low cost, allowing future coverage of an even greater proportion of patients.

In the current study, we used recurrence specimens to generate our list of trackable mutations, rather than pre-transplant specimens, which would perhaps better simulate real-world use of the assay. This was done for two reasons. First, we did not have access to pre-transplant samples from 6/30 patients, who either transferred their care from other centers or else did not have samples collected and saved from those time points, a particular problem prior to routine NGS profiling. Second, while the assay presented here includes surveillance only for pre-designated mutations at particular positions, future versions of this assay or a similar assay validated across wider areas of genomic territory following further refinement would permit identification of *de novo* variants not previously seen prior to transplant, making this point essentially moot. However, to assess the impact of this bias on our results, we re-analyzed our MRD data using only variants identified in pre-transplant samples. Pre-transplant mutational data/samples were available for 24 RG patients. Consistent with previously published reports, mutations were largely retained in post-relapse compared with pre-transplant specimens. Discounting pre-transplant mutations not seen at relapse, using only mutations identified prior to transplant would have reduced the number of realistically detectable mutations from an average 2.53 per patient to 1.7, though all patients would have retained at least one trackable marker. Despite this reduction, removal of post-transplant variants would have led to failure of MRD detection in only one of our 24 patients (RG29). In our original analysis, we detected both of this patient’s *WT1* mutations (seen only at recurrence) whereas the *FLT3* internal tandem duplication (ITD) present at both time points was undetectable.

In our study, we observed a fairly high MRD detection rate in samples collected 61–80 days prior to relapse, even though detected VAFs were lower on average compared with variants detected during time windows closer to recurrence. This is likely explained by an over-abundance in indels that happened to be detected at VAFs below 0.1% in this group. The disappearance of any MRD signal beyond 80 days pre-relapse is generally indicative of rapid exponential growth of recurrent clones. This fits with previously described data indicating AML recurrence doubling times as low as 11 days [34]. Clearly, methods that can detect residual AML at much earlier time points would be desirable, however boosting lead-time by more than a few months would likely require increasing the analytical sensitivity of the assay by multiple logs.

The most promising aspect of the NGS-MRD assay from the standpoint of potential clinical implementation is its specificity. In patients who never experienced a relapse, we never detected any sign of their original mutations in blood or bone marrow samples taken from a well-engrafted time point (n = 13 mutations). Likewise, no mutant signal was detected in our relapsed patients from time-points further removed in time from relapse (>80 days). While marrow histology and STR-PCR can each suffer from specificity issues, this NGS-MRD approach appears to have a specificity approaching 100%. For every samples used in this study the MRD status determined by STR, NGS-MRD assay and Multicolor Flow Cytometry (MFC) (when available) is shown in Table 2.

**Table 2:** MRD status for all the samples used in this study determined by three different assays.

Patient ID	Sample type	Days before recurrence	MRD status determined by		
			STR-PCR	NGS-MRD	MFC
RG01	BM	21	Negative	Positive	Negative
RG01	PB	21	Negative	Negative	Assay not performed
RG02	BM	38	Negative	Positive	Assay not performed
RG02	BM	119	Negative	Negative	Negative
RG03	BM	44	Negative	Positive	Negative/indeterminate
RG04	BM	31	Negative	Positive	Assay not performed
RG04	PB	31	Negative	Positive	Assay not performed
RG05	BM	41	Negative	Negative	Assay not performed
RG06	BM	49	Negative	Negative	Assay not performed
RG07	BM	43	Negative	Positive	Negative
RG07	PB	43	Negative	Negative	Assay not performed
RG08	BM	50	Negative	Positive	Assay not performed
RG08	PB	50	Negative	Negative	Assay not performed
RG08	BM	98	Negative	Negative	Assay not performed
RG08	PB	98	Negative	Negative	Assay not performed
RG09	BM	56	Negative	Negative	Assay not performed
RG10	BM	60	Negative	Negative	Negative
RG11	BM	72	Negative	Positive	Assay not performed
RG11	BM	136	Negative	Negative	Assay not performed
RG12	BM	61	Negative	Positive	Assay not performed
RG12	PB	61	Negative	Negative	Assay not performed
RG12	BM	117	Negative	Negative	Assay not performed
RG13	BM	95	Negative	Negative	Assay not performed
RG14	BM	99	Negative	Negative	Assay not performed
RG15	PB	84	Negative	Negative	Assay not performed
RG16	PB	32	Negative	Positive	Assay not performed
RG16	BM	77	Negative	Positive	Assay not performed
RG16	PB	77	Negative	Positive	Assay not performed
RG16	BM	105	Negative	Negative	Assay not performed
RG17	BM	28	Negative	Negative	Assay not performed
RG18	PB	27	Negative	Negative	Assay not performed
RG18	BM	69	Negative	Positive	Negative
RG18	PB	69	Negative	Negative	Assay not performed
RG19	PB	48	Negative	Positive	Assay not performed
RG19	BM	76	Negative	Positive	Assay not performed
RG19	BM	147	Negative	Negative	Assay not performed
RG20	PB	49	Negative	Positive	Assay not performed
RG20	PB	204	Negative	Negative	Assay not performed
RG21	BM	79	Negative	Negative	Assay not performed
RG22	BM	69	Negative	Positive	Assay not performed
RG22	BM	147	Negative	Negative	Assay not performed
RG22	PB	147	Negative	Negative	Assay not performed
RG23	PB	21	Negative	Positive	Assay not performed
RG23	BM	86	Negative	Negative	Assay not performed
RG24	BM	32	Negative	Negative	Assay not performed
RG25	BM	46	Negative	Positive	Assay not performed
RG25	BM	105	Negative	Negative	Negative
RG26	BM	44	Negative	Negative	Assay not performed
RG27	PB	32	Negative	Positive	Assay not performed
RG27	BM	66	Negative	Positive	Assay not performed
RG28	PB	61	Negative	Positive	Assay not performed
RG28	PB	117	Negative	Negative	Assay not performed
RG29	PB	52	Negative	Negative	Assay not performed
RG29	BM	91	Negative	Negative	Assay not performed
RG30	PB	35	Negative	Positive	Assay not performed
RG30	BM	74	Negative	Positive	Negative

The mutations surveyed in this retrospective analysis included those that are characteristically specific for AML (e.g. *NPM1*, *FLT3*) as well as mutations that are seen in both AML as well as pre-malignant clonal proliferations (e.g. *DNMT3A*). In the pre-transplant setting, there is substantial concern about using genes such as *DNMT3A* as MRD markers, because mutations in pre-malignant clonal proliferations are often seen at appreciable levels in otherwise successfully treated patients while AML-specific mutations in the same patient become undetectable[29,30]. Our data suggests that detection of any original somatic pathogenic mutation in a post-transplant sample should be extremely concerning for relapse. In the non-recurrence group, none of the patients’ original mutations in common CHIP-associated genes were detected. Likewise, any mutations in such genes that were detected in the recurrence population in pre-relapse time points all disappeared if tested in additional time-points further removed from relapse. Thus, the post-transplant scenario allows for more clinically straightforward mutation-based MRD surveillance compared with patients treated with chemotherapy alone.

Though we did note comparable sensitivity for relapse in PB (50%) vs. BM samples (53.6%) during this 20–100 day pre-relapse analysis, we did observe a marked difference in detected VAFs between time-matched PB and BM samples. There was also one patient RG18 that was MRD negative via analysis of PB sample at 27 days pre-relapse but whose BM sample was positive for MRD earlier, at day 69 (* in Fig 5B). These observations suggest that BM if available should be the preferred specimen type for MRD analysis. However, PB is more easily accessible and can be sampled far more frequently at lower cost in both dollars and comfort. Based on our results, it is likely that despite some degree of sensitivity gap, monthly NGS MRD PB monitoring may be overall more clinically effective than similar analysis of BM, which can only be sampled every 3–6 months. Future prospective clinical studies involving post-transplant patients should include this assay or a similar assay during the post-transplant period to allow for a cost-benefit analysis of this methodology compared with standard monitoring methods.

In conclusion, our results indicate that high sensitivity NGS MRD mutation surveillance incorporating error-correcting technology is more effective at detecting relapse in the post-transplant setting compared with STR PCR, hematopathologic and typical MFC analyses. During the retrospective time-frame, multi-color flow cytometry was only performed for cases with >1% blasts. Further, due to the multi-year retrospective nature of this analysis, we were unable to compare our results against newer highly-multiplexed flow cytometric methods only recently introduced in our laboratory. We will undertake this in a follow-up study. Based on the results of this study, assays covering expanded genomic territory for mutation detection (such as the NGS-MRD assay described here) should be actively investigated on a prospective basis to assess their suitability for integration into standard post-transplant surveillance procedures.

## Supporting information

S1 Table Patient characteristics. (DOCX)

S2 Table NRG samples at a well–engrafted time points MRD negative. (DOCX)

S3 Table The 59 trackable pathogenic variants (47 in RG, 9 in NRG and 3 excluded) and their distribution in the patients in this study. (DOCX)
