# Supplementary material for: Measurable residual disease monitoring for patients with acute myeloid leukemia following hematopoietic cell transplantation using error corrected hybrid capture next generation sequencing
Source: PLoS One. 2019 Oct 28;14(10):e0224097. doi: 10.1371/journal.pone.0224097 (PMC6816574; doi:10.1371/journal.pone.0224097)
Supplement: S1 Table — (DOCX) [file pone.0224097.s001.docx]

# S1 Table. Patient characteristics

| Median Age at Dx (IQR) | 51 (44-58) |
| --- | --- |
| Sex (% female) | 42.5 |
| ELN-2017 Risk Group |  |
| % Favorable | 15.0 |
| % Intermediate | 45.0 |
| % Unfavorable | 40.0 |
| % de novo AML | 67.5 |
| % Primary Refractory | 45.0 |
| Median Lines of Therapy Pre-SCT | 1.6 |
| % Morphologic CR Pre-Transplant | 72.5 |
| Graft Source |  |
| % MRD | 50 |
| % MUD | 30 |
| % Haplo-cord | 20 |
| Myeloablative Conditioning | 17.5 |
| Post-transplant DLI | 37.5 |
