# Supplementary material for: Measurable residual disease monitoring for patients with acute myeloid leukemia following hematopoietic cell transplantation using error corrected hybrid capture next generation sequencing
Source: PLoS One. 2019 Oct 28;14(10):e0224097. doi: 10.1371/journal.pone.0224097 (PMC6816574; doi:10.1371/journal.pone.0224097)
Supplement: S2 Table — (DOCX) [file pone.0224097.s002.docx]

# S2 Table. NRG samples at a well –engrafted time points MRD negative.

| **Patient ID** | **Source** | **Gene** | **Variant** | **Test date** | **MRD Status** |
| --- | --- | --- | --- | --- | --- |
| NRG01 | BM | *DNMT3A* | p.Arg882His | 3/14/17 | Negative |
| NRG01 | BM | *NPM1* | p.Trp288Cysfs*12 | 3/14/17 | Negative |
| NRG01 | BM | *FLT3* | c.1772_1837+39dup | 3/14/17 | Negative |
| NRG02 | PB | *NPM1* | p.Trp288Cysfs*12 | 9/25/17 | Negative |
| NRG02 | PB | *ASXL1* | p.G587Rfs*32 | 9/25/17 | Negative |
| NRG03 | BM | *NRAS* | p.Gly12Ala | 1/13/16 | Negative |
| NRG03 | BM | *NPM1* | p.Trp288Cysfs*12 | 1/13/16 | Negative |
| NRG03 | PB | *NRAS* | p.Gly12Ala | 1/13/16 | Negative |
| NRG03 | PB | *NPM1* | p.Trp288Cysfs*12 | 1/13/16 | Negative |
| NRG04 | PB | *FLT3* | p.Gln580Leufs*3 | 7/28/15 | Negative |
| NRG05 | PB | *NPM1* | outside case | 10/3/17 | Negative |
| NRG06 | PB | *NPM1* | p.Trp288Cysfs*12 | 10/31/17 | Negative |
| NRG06 | PB | *SRSF2* | p.Pro95Leu | 10/31/17 | Negative |
| NRG07 | BM | *TP53* | p.Ser215Gly | 7/5/16 | Negative |
| NRG07 | PB | *TP53* | p.Ser215Gly | 7/5/16 | Negative |
| NRG08 | BM | *FLT3* | c.1837+18_1837+19ins66 | 12/29/15 | Negative |
| NRG08 | BM | *FLT3* | p.Thr582_Leu601dup | 12/29/15 | Negative |
| NRG09 | BM | *SF3B1* | p.Lys666Met | 4/12/2017 | Negative |
| NRG09 | BM | *FLT3* | p.Asp835Tyr | 4/12/2017 | Negative |
| NRG09 | PB | *SF3B1* | p.Lys666Met | 4/12/2017 | Negative |
| NRG09 | PB | *FLT3* | p.Asp835Tyr | 4/12/2017 | Negative |
