# Supplementary material for: Measurable residual disease monitoring for patients with acute myeloid leukemia following hematopoietic cell transplantation using error corrected hybrid capture next generation sequencing
Source: PLoS One. 2019 Oct 28;14(10):e0224097. doi: 10.1371/journal.pone.0224097 (PMC6816574; doi:10.1371/journal.pone.0224097)
Supplement: S3 Table — (DOCX) [file pone.0224097.s003.docx]

**S3 Table. The 59 trackable pathogenic variants (47 in RG, 9 in NRG and 3 excluded) and their distribution in the patients in this study.**

| **Chromosome** | **Start Position** | **Gene** | **Variant** | **Samples** |
| --- | --- | --- | --- | --- |
| chr2 | 25457242 | *DNMT3A* | p.Arg882His | RG04, RG06, |
| chr2 | 25457243 | *DNMT3A* | p.Arg882Cys | RG01, RG13 |
| chr2 | 25457176 | *DNMT3A* | p.Pro904Leu | RG11 |
| chr13 | 28608222 | *FLT3* | p.Arg595_Glu611dup | RG10 |
| chr13 | 28602340 | *FLT3* | p.Asn676Lys | RG29 |
| chr13 | 28608274 | *FLT3* | p.Asp593_Phe594ins9 | RG14 |
| chr13 | 28608254 | *FLT3* | p.Asp600_Leu601ins10 | RG25 |
| chr13 | 28608254 | *FLT3* | p.Asp600_Leu601ins7 | RG04 |
| chr13 | 28592642 | *FLT3* | p.Asp835His | RG16,RG23,RG30 |
| chr13 | 28608260 | *FLT3* | p.Glu598_Tyr599ins17 | RG05 |
| chr13 | 28608220 | *FLT3* | p.Glu611_Phe612ins17 | RG3, RG26 |
| chr13 | 28608224 | *FLT3* | p.Gly583_Leu610dup | RG01 |
| chr13 | 28592635 | *FLT3* | p.Ile836del | RG15 |
| chr13 | 28608246 | *FLT3* | p.Phe594_Trp603dup | RG28 |
| chr13 | 28608245 | *FLT3* | p.Phe612_Gly613ins23 | RG11 |
| chr13 | 28608218 | *FLT3* | p.Phe612_Gly613ins29 | RG24 |
| chr13 | 28608236 | *FLT3* | p.Pro606_Arg607ins27 | RG24 |
| chr13 | 28608222 | *FLT3* | p.Thr582_Glu611dup | RG6 |
| chr13 | 28608218 | *FLT3* | p.Val579_Trp603dup | RG11 |
| chr13 | 28608277 | *FLT3* | p.Val592_Asp593insGluAla  LeuGlyGlySer | RG12 |
| chr2 | 209113113 | *IDH1* | p.Arg132Cys | RG08 |
| chr2 | 209113112 | *IDH1* | p.Arg132His | RG13, RG18 |
| chr15 | 90631934 | *IDH2* | p.Arg140Gln | RG03, RG04, RG20, RG26 |
| chr9 | 5073770 | *JAK2* | p.Val617Phe | RG27 |
| chr12 | 25398284 | *KRAS* | p.Gly12Asp | RG02 |
| chr12 | 25380276 | *KRAS* | p.Gln61Leu | RG22 |
| chr5 | 170837543 | *NPM1* | p.Trp288Cysfs*12 | RG01,RG02,RG04,RG05,RG06,RG09,RG10,RG13,  RG14,RG16,RG20,RG25,  RG29,RG30 |
| chr1 | 115258747 | *NRAS* | p.Gly12Ala | RG21 |
| chr2 | 198266834 | *SF3B1* | p.Lys700Glu | RG27 |
| chr17 | 7578389 | *TP53* | p.Arg181Cys | RG27 |
| **Chromosome** | **Start Position** | ***Gene*** | **Variant** | **Samples** |
| chr17 | 7578406 | *TP53* | p.Arg175His | RG15 |
| chr17 | 7577114 | *TP53* | p.Cys275Tyr | RG21 |
| chr17 | 7579332 | *TP53* | p.Ala119Profs*4 | RG07 |
| chr21 | 44524456 | *U2AF1* | p.Ser34Phe | RG19 |
| chr11 | 32417909 | *WT1* | p.Ala382fs | RG14 |
| chr11 | 32417909 | *WT1* | p.Ala382Serfs*7 | RG16 |
| chr11 | 32417907 | *WT1* | p.Ala387Valfs*4 | RG12 |
| chr11 | 32417945 | *WT1* | p.Arg369Glyfs*6 | RG17 |
| chr11 | 32417947 | *WT1* | p.Arg369Serfs*16 | RG28 |
| chr11 | 32417944 | *WT1* | p.Arg370Alafs*16 | RG02 |
| chr11 | 32413578 | *WT1* | p.Arg458* | RG29 |
| chr11 | 32413532 | *WT1* | p.His473Profs*4 | RG23 |
| chr11 | 32417913 | *WT1* | p.Arg380Leufs*72 | RG25 |
| chr11 | 32417873 | *WT1* | p.Leu378Argfs*5 | RG24 |
| chr11 | 32417909 | *WT1* | p.Pro372Glyfs*8 | RG24 |
| chr11 | 32417924 | *WT1* | p.Thr377Aspfs*8 | RG28 |
| chr11 | 32417899 | *WT1* | p.Thr385Profs*74 | RG30 |
| chr21 | 36252869 | *RUNX1* | p.Gly165Cys | RG19. High noise.Not included in analysis |
| chr11 | 32417910 | *WT1* | p.Ser381* | RG19 High noise.Not included in analysis |
| chr17 | 74732959 | *SRSF2* | p.Pro95His | RG03,RG26 High noise.Not included in analysis |
| chr20 | 31022270 | *ASXL1* | p.Gly587Argfs*32 | NRG02 |
| chr13 | 28608179 | *FLT3* | NM_004119.2:c.1772_1837+39dup | NRG01 |
| chr13 | 28608200 | *FLT3* | NM_004119.2:c.1837+18_1837+19ins66 | NRG08 |
| chr13 | 28592642 | *FLT3* | p.Asp835Tyr | NRG09 |
| chr13 | 28608317 | *FLT3* | p.Gln580Leufs*3 | NRG04 |
| chr13 | 28608253 | *FLT3* | p.Thr582_Leu601dup | NRG08 |
| chr2 | 198267360 | *SF3B1* | p.Lys666Met | NRG09 |
| chr17 | 74732959 | *SRSF2* | p.Pro95Leu | NRG06 |
| chr17 | 7578206 | *TP53* | p.Ser215Gly | NRG07 |
